# Augmentation of Pectoral Fin Teratogenicity by Thalidomide in Human Cytochrome P450 3A-Expressing Zebrafish

**DOI:** 10.3390/ph16030368

**Published:** 2023-02-28

**Authors:** Wenjing Dong, Ippo Akasaka, Akifumi Komiyama, Tatsuro Nakamura, Naohiro Mizoguchi, Tasuku Nawaji, Shinichi Ikushiro, Makoto Kobayashi, Hiroki Teraoka

**Affiliations:** 1 School of Veterinary Medicine, Rakuno Gakuen University, 582, Bunkyodai-Midorimachi, Ebetsu, Hokkaido 069-8501, Japan; 2 Chemicals Evaluation and Research Institute, Japan (CERI), 3-2-7, Miyanojin, Kurume, 839-0801, Fukuoka, Japan; 3 Department of Biotechnology, Faculty of Engineering, Toyama Prefectural University, 5180, Kurokawa, Imizu 939-0398, Toyama, Japan; 4 Department of Molecular and Developmental Biology, Faculty of Medicine, University of Tsukuba, Tsukuba 305-8575, Ibaraki, Japan

**Keywords:** CYP1A1, CYP3A4, CYP3A7, human, teratogenicity, thalidomide, zebrafish

## Abstract

The pharmacological and toxicological effects of active metabolites of enzymes including cytochrome P450 (CYP) are important. While it has been believed for a long time that thalidomide causes characteristic limb malformation only in rabbits and primates including humans, the involvement of their CYP3A subtypes (CYP3As) has been suggested. Recently, however, it was reported that zebrafish were sensitive to thalidomide, showing defects of pectoral fins, homologous organs of forelimbs in mammals, as well as other deformities. In this study, we prepared human CYP3A7 (hCYP3A7)-expressing zebrafish (F0) using a transposon system. Thalidomide caused pectoral fin defects and other malformations including pericardial edema in hCYP3A7-expressing embryos/larvae but not in wild-type and hCYP1A1-expressing embryos/larvae. Thalidomide also reduced the expression of fibroblast growth factor 8 in pectoral fin buds in only hCYP3A7-expressing embryos/larvae. The results suggest the involvement of human-type CYP3A in thalidomide teratogenicity.

## 1. Introduction

Zebrafish have been widely used in toxicological and pharmacological fields for about two decades. They were originally used as model animals for developmental biology [1]. Zebrafish embryos are annually available, small in size and transparent for a relatively long time, allowing chemical challenge and phenotype observation under a conventional microscope in a plastic dish for cultured cells. Their small size is also convenient for examining the effects of small amounts of precious chemicals and harmful wastes. According to regulations in the European Union, zebrafish are out of regulation as animal experiments until they have acquired self-feeding ability that is completed by 120 h post fertilization (hpf) [2]. Thus, zebrafish are now being used for the high-throughput screening of chemicals including medical drug candidates and possible environmental pollutants such as pesticides, especially for toxicological testing [3,4,5]. However, species differences between zebrafish and higher vertebrates should always be considered.

Cytochrome P450 (CYP) enzymes (CYPs) are a superfamily of enzymes that are involved in many metabolic reactions including the detoxication of xenobiotic substances such as medical drugs as well as other active substances such as steroid hormones [6]. It is also known that CYPs are involved in the conversion of prodrugs to active forms and in the bioactivation of promutagens and procarcinogens [7]. Harmful metabolites produced through the bioactivation by CYPs as well as other metabolic enzymes are also involved in many toxicological responses including developmental toxicity [6,8,9]. Thus, it is believed that species differences in CYPs are one of the major causes of different reactions to chemicals in each species [10].

Thalidomide was originally marketed as a sedative and preventive medicine for morning sickness in pregnant women. However, around 1960, it was found that thalidomide caused limb defects called phocomelia and other birth defects in the eyes, ears, internal organs, heart and vascular system [11]. It is well known that thalidomide has two optical isomers with a mirror-symmetric spatial structure. The R-enantiomer is known to inhibit pregnancy reactions and sedation, while the *S*-enantiomer is known to be teratogenic [12,13]. Although many researchers worked on the causes of these characteristic deformities caused by thalidomide, the mechanism of toxicity was not known for a long time mainly due to species differences in sensitivity to thalidomide. Phocomelia-like limb defects caused by thalidomide were observed in primates including humans and rabbits but not in rodents and other major experimental animals [14]. For this reason, rabbits are still used in developmental toxicology testing in addition to their use in research. Thalidomide also has immunomodulatory, anti-inflammatory and antiangiogenic effects [15]. Thus, thalidomide has been approved again as an anticancer agent in many countries, although its use was banned in the early 1960s.

It is well known that oxidative metabolites of thalidomide are different in humans and other thalidomide-sensitive animals (non-human primates and rabbits) and thalidomide-insensitive animals such as rodents. 5-Hydroxythalidomide is the major oxidative metabolite of thalidomide in humans and rabbits, while the major oxidative metabolite is 5’-hydroxythalidomide in rodents [16]. Human CYP3A4 (hCYP3A4) and CYP3A5 (hCYP3A5) are involved in the conversion of thalidomide to 5-hydroxythalidomide [17,18]. Thalidomide induced hCYP3A4 and hCYP3A5 in BeWo cells, which are human placenta-derived cells [19]. hCYP2C19 also produced 5-hydroxythalidomide, while rCYP2C11, a dominant CYP2C subtype in male rats, produced 5′-hydroxythalidomide [20]. While CYP3A4 and hCYP3A5, accounting for about 30-40% of the total CYP content in the human liver, play a major role in the metabolism of xenogeneic organisms, transcripts of hCYP3A7, but not those of hCYP3A4/5, were detected as early as day 50 of gestation as fetal liver-specific CYP subtypes. Protein and specific activity of hCYP3A7 were also detected in the placenta and the fetal liver of humans [21,22].

In 2010, a group of Handa reported pectoral fin defects caused by thalidomide in developing zebrafish as well as wing defects in chick embryos [23]. More importantly, they also identified cereblon (CRBN), a component of ubiqutinase, as a possible target of thalidomide. They suggested that the binding of thalidomide to the complex of cullin4 (CUL4) and CRBN caused the degradation of some target proteins, SALL4, ΔNp63, TAp63 and PLZF, resulting in limb defects [15,24,25]. Effects of thalidomide on transcripts of some genes that might be related to the development of forelimbs or homologous organs have also been reported. Thalidomide inhibited the expression of fibroblast growth factor 8 (fgf8) without having an effect on sonic hedgehog (shh) expression in pectoral fin buds of zebrafish [23]. Similarly, the expression of fgf8 and 10 was generally reduced by thalidomide exposure in wing buds of chickens and forelimb buds of rabbits [14,23,26,27]. Recently, Miyagawa and co-workers reported specific formation of an SALL4-CRBN complex and SALL4-specific degradation by 5-hydroxythalidomide, a major metabolite of thalidomide, but not by thalidomide, in humans and rabbits, while thalidomide induced the formation of an IKZF1–CRBN complex [28]. From these observations, they proposed that SALL4 degradation and IKZF1 degradation led to teratogenicity and immunomodulatory effects of thalidomide, respectively. Kazuki et al. [29] found that thalidomide induced the malformation of limb buds in cultured whole embryos of transchromosomic mice containing a human CYP3A cluster, indicating that the exogenous expression of human CYP3As could reproduce thalidomide teratogenicity in animals other than rabbits and primates. These observations suggest that human-type CYP3As might be involved in thalidomide teratogenicity including limb defects.

Recently, however, it was reported that conventional aqueous exposure to thalidomide has little effect on the normal development of zebrafish [30]. The purpose of this study was to prepare an acute zebrafish model expressing human CYP3As, in which pectoral fin defects and other malformations were caused by conventional waterborne exposure to thalidomide, for toxicological studies.

## 2. Results

### 2.1. EGFP and hCYP3A7 Expression in hCYP3A7 Plasmid-Injected Zebrafish

Observations under stereo and inverted microscopes showed that zebrafish that had been injected with a mixture of hCYP3A7/EGFP plasmid and capped mRNA (cmRNA) of transposase at the one-cell stage (hCYP3A7 embryos) appeared to develop normally until 120 hpf.

**Figure 1:**
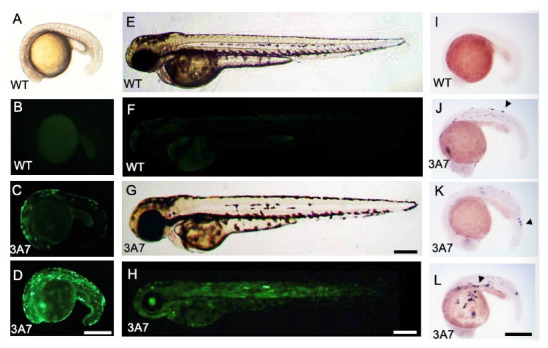
EGFP and CYP3A7 expression in hCYP3A7/EGFP-expressing zebrafish. A pT2A plasmid containing hCYP3A7/EGFP (3A7: **C**,**D**,**G**,**H**,**J**–**L**) was injected together with transposase cmRNA into 1-cell stage embryos. (**A**–**D**): 17 hpf, (**E**–**H**): 50 hpf. EGFP fluorescence in wild-type (WT) zebrafish (**B**,**F**) and that in hCYP3A7-expressing zebrafish (hCYP3A7 zebrafish, 3A7) (**C**,**D**,**H**) are shown. Image (**A**) is a bright field image of a wild-type embryo. Images (**E**,**G**) are bright field images of (**F**,**H**), respectively. CYP3A7 mRNA expression was observed at 23 hpf by WISH (**I**–**L**). Arrowheads indicate examples of positive signals in **J**–**L**. Scale bars = 200 µm.

Strong but mosaic-like EGFP expression was observed in hCYP3A7 zebrafish during development at least after 17 hpf compared to that in wild-type zebrafish (Figure 1B–D,F,H).

hCYP3A7 transcripts were detected in hCYP3A7 zebrafish but not in wild-type zebrafish by whole-mount in situ hybridization (WISH) (Figure 1I–L). Expression patterns of hCYP3A7 transcripts were also mosaic-like, being consistent with EGFP fluorescence (Figure 1C,D). Similar to EGFP fluorescence (Figure 1C,D), the expression pattern and intensity of positive signals of hCYP3A7 by WISH were quite variable for zebrafish during development (Figure 1J–L).

### 2.2. Effects of Thalidomide on hCYP3As-Expressing Zebrafish during Development

**Figure 2:**
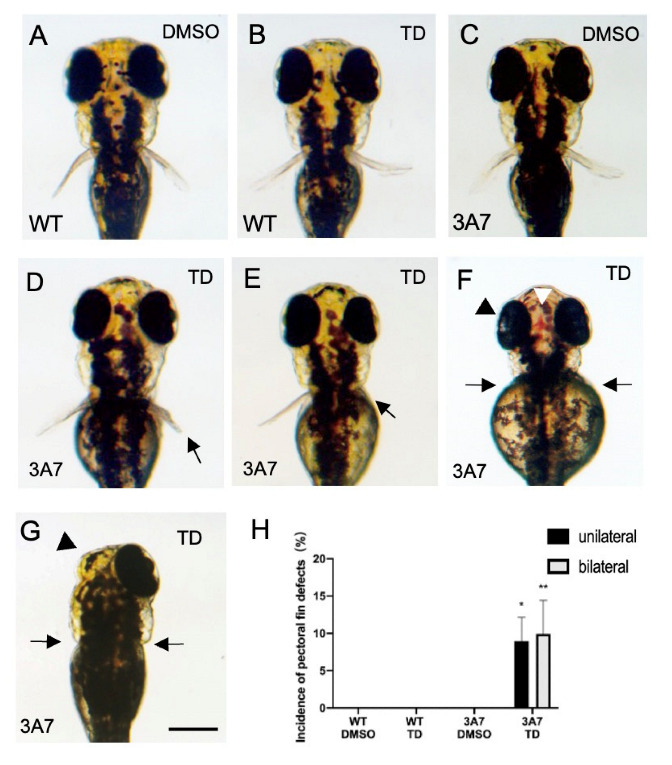
Effects of thalidomide on pectoral fin development of hCYP3A7-expressing zebrafish. Embryos/larvae of wild-type (WT) zebrafish (**A**,**B**) and hCYP3A7-expressing zebrafish (3A7) (**C**–**G**) were exposed to 200 μM thalidomide (TD) or 0.02% DMSO as a vehicle from 17 hpf to 72 hpf. Arrows in (**D**–**G**) indicate pectoral fin malformation. The white arrowhead in (**F**) indicates bleeding in the dorsal head region. Black arrowheads show a smaller eye (**F**) and loss of an eye (**G**). Scale bar = 200 µm. A summarized graph is presented in H (*n* = 6, 8–26 zebrafish for each case). * *p* < 0.05, ** *p* < 0.01.

**Figure 3:**
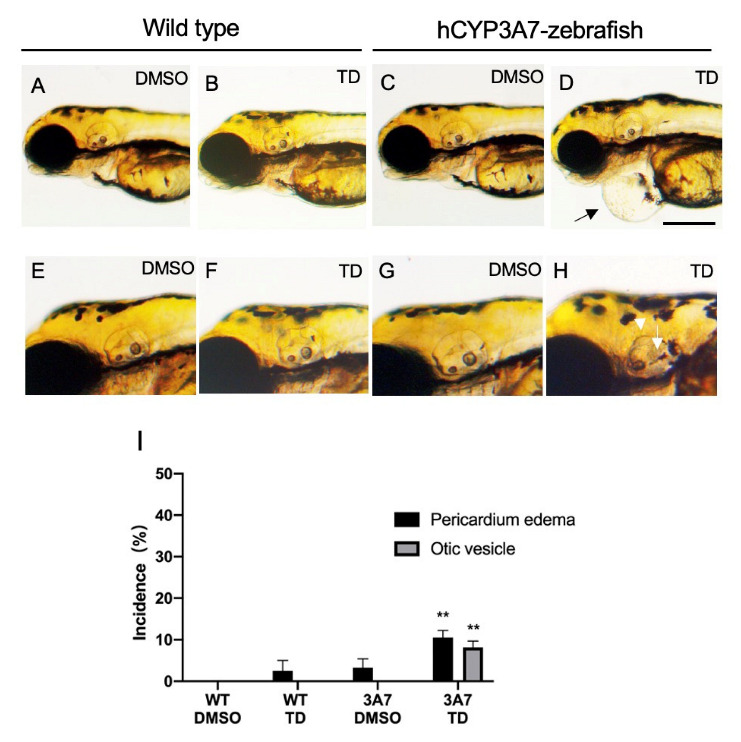
Effects of thalidomide on pericardial edema and otic vesicles of hCYP3A7-expressing zebrafish. Embryos/larvae of wild-type (WT) zebrafish (**A**,**B**,**E**,**F**) and hCYP3A7-expressing zebrafish (3A7) (**C**,**D**,**G**,**H**) were exposed to 200 μM thalidomide (TD) or 0.02% DMSO as a vehicle from 17 hpf to 72 hpf. The arrow in D indicates severe pericardial edema. The arrow and arrowhead show loss of a large otolith (saccule) and round otic vesicle, respectively. Scale bar = 200 µm. Summarized data are presented as a graph in (**I**) (*n* = 4, 8–26 zebrafish for each case). ** *p* < 0.01.

No malformations were observed in wild-type zebrafish that were exposed to thalidomide (200 µM) from 17 hpf to 72 hpf (Figure 2 and Figure 3). A higher concentration of thalidomide (400 µM, 0.04% DMSO) also had no effect on the development of zebrafish until 72 hpf.

When zebrafish were exposed to 200 µM thalidomide from 17 hpf to 72 hpf, pectoral fin defects were observed in hCYP3A7 zebrafish (Figure 2D–G: C being a vehicle control) but not in wild-type zebrafish at 72 hpf (Figure 2B: A being a vehicle control). Pectoral fin defects included shortening and complete loss unilaterally or bilaterally (Figure 2D–G). Bilateral and unilateral pectoral fin defects occurred at a similar rate in hCYP3A7 zebrafish (Figure 2H). Similar malformations including pectoral fin defects were also found in hCYP3A7 zebrafish (*n* = 53) that were exposed from 24 hpf (7.5% for pectoral fin defects, 13.2% for pectoral fin shortening, 15.1% for severe edema, 11.3% for eye malformation).

Thalidomide also induced other malformations including pericardial edema (Figure 3D) and defects of otic vesicles including otoliths (Figure 3H) in hCYP3A7 zebrafish but not in wild-type zebrafish (Figure 3A,B,E,F). Smaller eyes and loss of eyes caused by thalidomide were also found in some hCYP3A7 larvae (Figure 2F,G). The incidences of both malformations were about 10% or less (Figure 3H).

A lower concentration of thalidomide (100 µM) did not cause malformation of pectoral fins even in hCYP3A7 zebrafish, while one larva (1 of 33) showed severe edema. When thalidomide (200 µM) exposure was started from 30 hpf, thalidomide did not cause any malformation in wild-type and hCYP3A7 zebrafish except for one larva showing loss of both otoliths in the right otic vesicle only (*n* = 30).

Some hCYP3A7 larvae showed pectoral fin defects and pericardial edema at the same time, while most of the larvae with pectoral fin defects did not have severe edema or a reduction in blood flow through the aorta and cardinal vein around the pectoral fins (Table S1). As an exception, one larva showed bleeding in the dorsal head (Figure 2F). However, a significant change in blood flow around the pectoral fins was not observed by a stereomicroscope in this larva.

Similar malformations including pectoral fin defects were also found in hCYP3A4-expressing zebrafish (hCYP3A4 zebrafish) exposed to 200 µM thalidomide (Figure S1). Severe pericardial edema caused by thalidomide was not found even in hCYP3A4 zebrafish by 72 hpf (*n* = 35). Remarkable circulation failure including reduction in blood flow through major blood vessels, especially around the pectoral fins, and infarction were not observed at 48 hpf and 72 hpf in hCYP3A4 embryos/larvae exposed to thalidomide.

### 2.3. Effects of Thalidomide on hCYP1A1-Expressing Zebrafish during Development

Mosaic-like EGFP fluorescence was observed in hCYP1A1/EGFP-expressing zebrafish (hCYP1A1-zebrafish) (Figure S2B). After exposure to 7-ethoxyresorufin, a specific substrate for CYP1A, strong red fluorescence of hydroxyresorufin, a metabolite of 7-ethoxresorufin, was detected in hCYP1A1-embryos/larvae but not in wild-type embryos/larvae (Figure S2D,F).

Bezo[a]pyrene (25 µM), the most well-known chemical for acquired toxicity by bioactivation through CYP1A, caused severe pericardial edema in hCYP1A1-larvae (20/22, 90.9%) but not in wild-type larvae (0/30) (Figure S2G,H).

**Figure 4:**
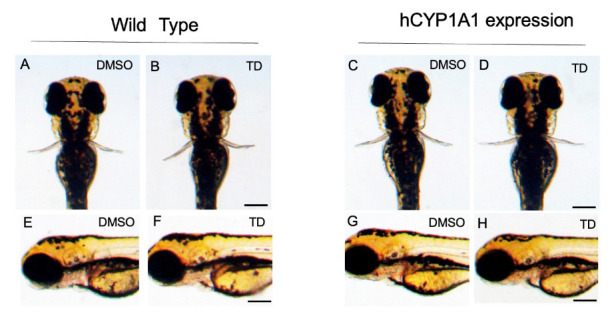
Effects of thalidomide on hCYP1A1-expressing zebrafish during development. Embryos/larvae of wild-type (WT) zebrafish and hCYP1A1-expressing zebrafish (hCYP1A1-zebrafish) were exposed to 200 μM thalidomide (TD) or 0.02% DMSO as a vehicle from 17 hpf to 72 hpf. (**A**,**B**, **E**,**F**): wild-type zebrafish. (**C**,**D**,**G**,**H**): hCYP1A1-zebrafish. Zebrafish in (**A**,**E**,**C**,**G**) were exposed to DMSO. Zebrafish in (**B**,**F**,**D**,**H**) were exposed to thalidomide. (**A**–**D**): dorsal views; (**E**–**H**): lateral views. Scale bars = 200 µm.

Unlike in the hCYP3A4 and hCYP3A7 zebrafish, thalidomide did not cause any malformation in hCYP1A1 zebrafish as well as in wild-type zebrafish by 72 hpf (Figure 4) (*n* = 20).

### 2.4. Effects of Thalidomide on the Expression of Some Genes Related to Pectoral Fin Development in hCYP3A7-Expressing Zebrafish

**Figure 5:**
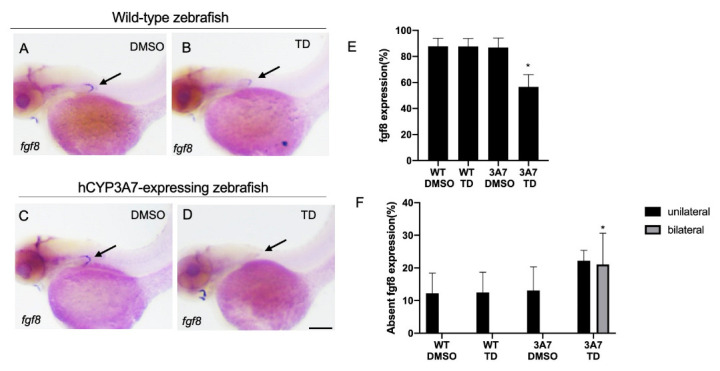
Thalidomide-induced reduction in fgf8 expression in pectoral fin buds of hCYP3A7-expressing zebrafish. Wild-type (WT) (**A**,**B**) and hCYP3A7-expressing embryos/larvae (CYP3A7) (**C**,**D**) were exposed to 200 µM thalidomide (TD) or 0.02% DMSO (DMSO) as a vehicle from 17 hpf until 48 hpf. Larvae were fixed at 48 hpf for whole-mount in situ hybridization with an fgf8 probe. Summarized data are presented as graphs in (**E**,**F**) (*n* = 3, 7–10 embryos for each case). (**E**,**F**) indicate percentages of fgf8 expression and percentages of unilateral and bilateral loss of fgf8 expression in the presence or absence of thalidomide. * *p* < 0.05. Scale bar =100 µm.

Whole-mount in situ hybridization (WISH) clearly showed the expression of fibroblast growth factor 8 (fgf8) in the possible apical ectodermal ridge (AER) of pectoral fin buds of most wild-type zebrafish larvae at 48 hpf (Figure 5A). Fgf8 transcripts were detected in most wild-type zebrafish regardless of thalidomide exposure, although about 10% of the embryos/larvae did not show fgf8 expression possibly due to technical failure (Figure 5E,F). In contrast, the percentage of hCYP3A7 embryos/larvae that showed fgf8 expression in pectoral fin buds was significantly decreased by thalidomide exposure compared to the percentage in a vehicle treatment group and wild-type groups with and without thalidomide exposure (Figure 5E). Both bilateral loss and unilateral loss of fgf8 in pectoral fin buds caused by thalidomide were observed in hCYP3A7 zebrafish, although the increase in the incidence of unilateral loss was not significant (Figure 5F).

Fgf8 expression in other regions including the midbrain–hindbrain boundary (MHB) and the hyoid can be recognized (Thisse et al., 2001 - Expression of the zebrafish genome during embryogenesis (NIH R01 RR15402). ZFIN Direct Data Submission) (Figure S3). Fgf8 expression in the hyoid was not affected by thalidomide in both wild-type and hCYP3A7 embryos/larvae (Figure S3B,D), while the expression of fgf8 in the MHB had almost disappeared in both wild-type and hCYP3A7 embryos/larvae in this stage.

Effects of thalidomide on the expression of sall4 (Figure S4A,B,E,F) and the expression of sonic hedgehog (shh) (Figure S4C,D,G,H) were also studied. Sall4 transcripts were found throughout the pectoral fin buds and shh transcripts were restricted to the posterior margin of the pectoral fin buds in zebrafish at 48 hpf [23,31]. The expression of sall4 and shh was hardly affected by thalidomide exposure in either wild-type or hCYP3A7 embryos/larvae.

## 3. Discussion

In this study, we prepared hCYP3A7-expressing zebrafish (F0) as well as hCYP3A4- and hCYP1A1-expressing zebrafish using a transposon system [32]. These zebrafish models can be used to evaluate the effects of chemicals on early development. Although Poon et al. [33] established a transgenic zebrafish model expressing hCYP3A4, the most important CYP subtype for the metabolism of medicine in the adult human liver, development of the primordia of most major organs including pectoral fins was almost completed before hCYP3A4 expression since they used a hepatocyte-specific promoter for hCYP3A4 expression in the liver [33]. There had been concern that the exogenous expression of CYP enzymes might affect the normal development of zebrafish since it is well known that CYP3A subtypes have wider active sites and can react with various substrates including endogenous active substances [34]. However, zebrafish expressing these hCYPs appeared to develop normally at least until 120 hpf. CYP3A7/EGFP expression in F0 embryos/larvae was mosaic-like and variable. Mosaic-like expression was very common in developing zebrafish when an EGFP plasmid was injected into early blastomeres [35]. This pattern of expression of hCYP3As and 1A1 might contribute to diverse malformations in addition to the absence of serious effects on the general development of zebrafish. While more homologous expression of hCYP3A7 in zebrafish may result in serious impacts, it is still feasible to generate transgenic zebrafish expressing this enzyme, as has been accomplished with rat CYP2E1-transgenic fish [36].

It was reported that thalidomide induced slight forelimb malformation in transchromosomic mice containing a cluster of hCYP3As without having any effect on wild-type mice [29]. That study suggested a role of hCYP3A-dependent activity in teratogenesis including forelimb defects caused by thalidomide. Thalidomide-induced malformations in pectoral fins and other organs in wild-type zebrafish have been found in many studies [15,23,37,38]. On the other hand, however, it was also reported that effects of thalidomide by simple soaking in a thalidomide solution were very weak [30], as was confirmed in the present study. Thalidomide also had minor effects on the development of embryos/larvae by usual immersion but caused malformations including pectoral fin defects and curvature of the spine by electroporation in medaka (*Oryzias latipes*) [39]. In this study, thalidomide exposure (200 and 400 µM) did not cause any malformation in wild-type zebrafish when exposure was started from 17 hpf or 24 hpf. Ito et al. [23] reported that waterborne exposure to thalidomide from 24 hpf caused malformation including pectora fin defects, although earlier exposure to thalidomide (within 3 hpf) was more effective. Additionally, defects of pectoral fins including bilateral and unilateral complete loss were clearly detected in hCYP3A7 and hCYP3A4 zebrafish larvae but not in wild-type and hCYP1A1 larvae, suggesting the specificity of CYP3A. Disorders of the heart, eyes and ears were observed in humans, primates and rabbits [40]. In this study, pericardial edema and defects of the otic vesicle, an organ identical to the mammalian ear, were also found in only hCYP3As-expressing larvae exposed to thalidomide. Although only round otic vesicles were reported by Ito et al. [23], defects of otoliths including loss were observed in hCYP3As zebrafish. Local expression of hCYP3A in the otic vesicle primordium in some embryos/larvae might account for the difference to the observations by Ito et al. [23]. Collectively, the observations suggested that human-type CYP3As are involved in thalidomide teratogenicity using a zebrafish model. It was reported that hCYP3As are involved in the conversion of thalidomide to 5-hydroxythalidomide [17,18]. Cultured whole embryos of mice expressing an hCYP3A cluster showed malformation of limb buds caused by thalidomide [29]. Taking all of these things into consideration, our results support the hypothesis that 5-hydroxythalidomide, which was converted from thalidomide by hCYP3As, caused malformations including pectoral fin defects, although we did not confirm hCYP3As-dependent production of 5-hydroxythalidomide. Exposure to a high concentration of thalidomide of more than 200 µM resulted in pectoral fin defects and other malformations even in hCYP3A7-expressing zebrafish. A high concentration of thalidomide was also required to cause developmental toxicity possibly due to lower absorption of thalidomide by embryos/larvae of medaka [39]. It was also suggested that zebrafish might be relatively insensitive to thalidomide compared to humans due to mutations of SALL4, a possible key gene in pectoral fin growth [41]. It is speculated that thalidomide might have significant effects when hCYP3A7 was expressed in or around primordial cells of organs and tissues. In other words, a high concentration of 5-hydroxythalidomide may be required around primordial cells for causing malformation. This speculation could also be the possible explanation for why thalidomide-induced malformation was highly variable, particularly for bilateral/unilateral shortening or loss of pectoral fins, in hCYP3A7 zebrafish larvae, since EGFP/hCYP3A7 expression was mosaic-like and variable.

Thalidomide-induced pericardial edema was found in hCYP3A4/7 embryos/larvae but not in wild-type embryos/larvae in this study. However, antiangiogenic effects of thalidomide in wild-type zebrafish have been reported [26,42]. Failure of angiogenic outgrowth was suggested as a cause of thalidomide-induced pectoral fin malformation in zebrafish [26]. Although the effects of thalidomide on angiogenic outgrowth in hCYP3A4/7 embryos/larvae were not studied with transgenic fish expressing blood vessel-specific fluorophores in this study, circulation failure, including accumulation of erythrocytes in some vessels (congestion), was rarely observed around pectoral fins in thalidomide-exposed hCYP3As-embryos/larvae as well as in wild-type embryos/larvae. Additionally, there were many hCYP3A7 expressing embryos/larvae with pectoral fin defects without edema or circulation failure. Paulissen et al. [43] reported that the formation of the primitive pectoral artery was completed at 40 hpf, while thalidomide had no effects on pectoral fin growth from 30 hpf in hCYP3A7 zebrafish, suggesting that the impact of thalidomide on developing zebrafish before 30 hpf was critical for the formation of pectoral fin defects. Thus, an additional mechanism other than failure of angiogenic outgrowth should be considered in thalidomide-induced pectoral fin defects in hCYP3As-zebrafish.

The pectoral fin is regarded as a homologous organ of the mammalian forelimb and avian wing [44]. We usually selected exposure from 17 hpf since expression of tbx5, an essential gene for pectoral limb bud formation, was confirmed in pectoral fin buds from 17 hpf [31]. However, a remarkable difference in the teratogenic effects of thalidomide when exposure was started at 17 hpf and when exposure was started at 24 hpf was not found. It has been established that fibroblast growth factors (fgfs) play an essential role in the development of forelimbs and pectoral fins regardless of the animal species [45]. Thalidomide inhibits fgf8 expression in pectoral fin buds of zebrafish (wild type) and wing buds of chickens [23,25]. We confirmed a reduction in fgf8 expression in pectoral fin buds in hCYP3A7 zebrafish but not in wild-type zebrafish in this study. The rate of loss of fgf8 expression was comparable to that of pectoral fin malformation. Bilateral loss and unilateral loss of fgf8 were also observed, coinciding with the bilateral/unilateral shortening or loss of pectoral fins in hCYP3A7 embryos/larvae exposed to thalidomide. Expression of fgf8 in the hyoid at 48 hpf was rarely affected by thalidomide in hCYP3A7 embryos/larvae, suggesting selective effects of thalidomide on pectoral fin buds but not direct inhibition of fgf8 transcription. Fgf8 is believed to be indispensable for forelimb growth in chickens and rabbits, which are thalidomide-sensitive species [25,46]. Thalidomide reduced the expression of fgf8 and fgf10 in these species, suggesting that these are major targets of thalidomide in defects of forelimbs and wings [14,27]. In zebrafish, however, an fgf8 mutant (acerebellar, ace) had loss of the MHB but had normal pectoral fins, whereas the functional disruption of fgf24, fgf10 and fgf16 resulted in the loss of pectoral fins [46,47]. It was suggested that the loss of fgf8 and fgf4 can lead to limb hypoplasia in mice [48,49]. Fgf24 is specific for zebrafish and is also necessary to initiate growth of pectoral fin buds [46]. Thus, fgf10, 16 and 24 should be studied as possible targets of thalidomide using hCYP3A7 zebrafish in a future study. While the role of fgf8 in pectoral fin defects caused by thalidomide is not always clear in zebrafish, our results showed a reduction in fgf8 expression in pectoral fin buds caused by thalidomide in hCYP3A7-expressing zebrafish, being consistent with findings for forelimb buds of rabbits and wing buds of chickens.

It is believed that shh directs pattern formation along the anterior/posterior axis of the limb expressed in the zone of polarizing activity (ZPA) [50]. Thalidomide had no effect on the expression of shh even in pectoral fin buds of hCYP3A7 zebrafish, confirming the results of a previous study in wild-type zebrafish [23,51]. On the other hand, it was reported that shh expression was not observed in pectoral fin buds of zebrafish with loss-of-function of fgf10, 16 and 24 [52]. Shh expression was not affected by thalidomide in this study, suggesting that fgf10, 16 and 24 might not be downregulated in pectoral fin buds by thalidomide. However, the situation might be different in zebrafish that lost these fgfs maternally and zygotically from the situation in zebrafish that were exposed to thalidomide from 17 hpf. Further studies are needed to confirm the target of thalidomide as a cause of pectoral fin malformation in zebrafish.

Sall4 is expressed in the mesenchyme of fin buds and is thought to be essential for pectoral fin development in zebrafish [31]. We found that thalidomide had no effect on the expression of sall4 mRNA in both wild-type embryos/larvae and hCYP3A7 embryos/larvae. It was reported that thalidomide promoted the degradation of SALL4 protein without affecting its transcripts [41]. Since it was also reported that 5-hydroxythalidomide degrades sall4 protein, our observation was not against the possible involvement of sall4 in thalidomide-induced pectoral fin malformation [25].

Although there is a very large number of CYP subtypes in zebrafish [53], the properties of metabolism are unclear for most zebrafish CYP subtypes (zCYPs). Zebrafish have zCYP3A65 and zCYP3C (1-4) [53]. zCYP3A65 showed 51% amino acid identity with hCYP -3A4 and is considered as a homologue of human CYP3A4 [53]. Transcripts of zCYP3A65 and zCYP3Cs begin to increase after 96 hpf, at which time the development of pectoral fins is almost completed [54]. There is no information on the metabolism of thalidomide by these CYP3 subtypes. Using caffeine and diclofenac, metabolic activities similar to those of hCYP1A2, hCYP2C9 and hCYP3A4/5 without depending on liver organogenesis (about 48 hpf) have been reported in developing zebrafish [54]. The metabolism of thalidomide in developing zebrafish before 30 hpf, a period that is critical for teratogenesis induced by thalidomide, should be clarified in future studies.

## 4. Materials and Methods

### 4.1. Chemicals

(*S*)-Thalidomide (TD) was obtained from Sigma-Aldrich (98% purity (Sigma, St. Louis, MO, USA) and Toronto Research Chemicals (Toronto, Ontario, Canada). Benzo[a]pyrene and 7-ethoxyresorufin were purchased from Sigma-Aldrich and Cayman Chemical (Ann Arbor, MI, USA), respectively. All other chemicals were commercially available products of special reagent grade.

### 4.2. Preparation of pT2A Vector and Capped RNA for Expression of Human CYPs

Expression of human CYP3A4, 7 and CYP1A1 into the zebrafish genome was carried out according to Kawakami [32]. A medaka transposon vector (pT2A vector) carries a medaka β-actin promoter containing human CYP3A4, 7- and CYP1A1-2A peptide-enhanced green fluorescent protein (EGFP) (pT2A/medaka β-actin-p-3xFlag-human CYP3As / CYP1A1-2A-EGFP) (Figure S5). cDNA was prepared with total RNA of humans (Human BioBank cDNA: Eastleigh, UK) using PrimeScript Reverse Transcriptase (Takara Bio, Otsu, Japan). The first PCR and nested PCR were carried out with Tks Gflex DNA polymerase (Takara Bio, Otsu, Japan) and KOD Dash (TOYOBO, Osaka, Japan), respectively, following the manufacturers’ instructions. The primer sequences used are listed in Table S2. Primers for the nested PCR were attached with restriction enzyme recognition sites for subcloning into the pT2A vector (Figure S5). Nested PCR products were subcloned into the T-vector (pTAC-2, BioDynamics, Tokyo, Japan) by TA cloning. After complete restriction of the pTAC-2 vector with restriction enzymes (SalI and XhoI) (New England Biolabs, Ipswich, MA, USA), the resultant open reading frame of human CYP3As / CYP1A1 was subcloned into the pT2A vector containing toll2 sites between 3xFlag and 2A peptides using a DNA ligation kit (Mighty Mix, Takara Bio). Capped mRNA (cmRNA) of transposase was transcribed in vitro with the mMESSAGE mMACHINE^TM^ SP6 Transcription Kit (Thermo Fisher, Waltham, MA, USA). The original pT2A vector and a transposase plasmid as a template (pCS2+transposase) were kind gifts from Dr. Kawakami.

### 4.3. Zebrafish Culture, Injection of a Vector Mixture and Thalidomide Exposure

Adult zebrafish were maintained at 28.5°C in a re-circulating aquatic habitat system under a 14 h:10 h (light:dark) photoperiod as previously reported [55]. Female and male adult zebrafish were mated at a ratio of 1:1, and the embryos were collected and maintained in Zebrafish Ringer solution (ZR solution) (38.7 mM NaCl, 1.0 mM KCl, 1.7 mM HEPES-NaOH pH 7.2, 2.4 mM CaCl_2_) at 28.5°C in an incubator.

One single blastomere of each one-cell stage zebrafish embryo was injected with a mixture of 10 ng/µL plasmid DNA and 10 ng/µL cmRNA of transposase in Ca^2+^-free ZR solution by using a fine glass needle connected to an automatic injector (IM-300: Narishige, Japan). EGFP-expressing embryos were selected under a fluorescent stereomicroscope (MZ10F, Leica Microsystems, Wetzlar, Germany) and manually dechorionized at 17 hpf as were wild-type embryos. Dead and malformed embryos were removed at 17 hpf. Normally developed embryos were incubated at 28.5 ℃ in 3.5 cm petri dishes (AGC Techno Glass, Yoshida, Japan) each containing 3 mL ZR solution, with 10–13 embryos per dish. Zebrafish were usually exposed to 200 µM thalidomide by adding 0.6 μL of 1 M (*S*)-thalidomide (thalidomide) stock solution (dimethylsulfoxide, DMSO) to 3 mL ZR solution. DMSO (0.02%) was used as a vehicle control since it was reported that DMSO inhibits CYP activity [56]. The thalidomide solution was exchanged every 24 hr up to 72 hpf. Morphology and circulation status were observed under a stereomicroscope (SZX-12, Olympus, Tokyo, Japan) and an inverted microscope (IX71, Olympus). Shortening of pectoral fins caused by thalidomide was determined according to Asatsuma-Okumura et al. [51] (Figure S6). Severity of pericardial edema was determined according to the position relative to the yolk (Figure S7). Edema and other malformations were double-checked by two authors.

### 4.4. Whole-Mount In Situ Hybridization (WISH)

To obtain probes, zebrafish sall4 and hCYP3A7 were amplified with cDNA of 120 hpf zebrafish larvae by PCR reaction with the primer sets listed in Table S3. This was followed by subcloning to the pTAC-2 vector and in vitro transcription with SP6 RNA polymerase (New England Biolabs, Ipswich, MA, USA). Fgf8 and shh plasmids were provided by Drs. Ingham [57] and Brand [47].

Upon reaching 17–50 hpf, zebrafish embryos were fixed in 4% (*w*/*v*) paraformaldehyde overnight. Whole-mount in situ hybridization (WISH) was carried out according to a previously reported method [54]. Embryos were hybridized with an antisense RNA probe of zebrafish fgf8, sall4, shh and hCYP3A7 overnight at 64 °C and then washed with three different concentrations of saline sodium citrate. The embryos were blocked with 2% blocking reagent (Sigma-Aldrich), followed by incubation with an anti-DIG antibody conjugated with alkaline phosphatase (Sigma-Aldrich) at 4 °C overnight. Color reaction was carried out by incubation with NBT/BCIP Solution (Sigma-Aldrich). Hybridization images were double-checked by two authors.

### 4.5. Statistics

Results are expressed as means ± SEM. Significant differences between two groups were determined by one-way ANOVA followed by the Tukey–Kramer test (*p* < 0.05). The analysis was carried out using Excel 2016 with Statcel (addin forms on Excel-4th ed.) (OMS Publishing, Tokyo, Japan).

## 5. Conclusions

The results showed that heterologous expression of hCYP3As in zebrafish augmented thalidomide-induced malformations including pectoral fin defects. Although the role of fgf8 in pectoral fin defects caused by thalidomide was not always clear, the common mechanism of thalidomide-induced limb malformation in rabbits was confirmed in pectoral fin buds of zebrafish. This is additional evidence for the involvement of hCYP3As in limb malformation caused by thalidomide. The zebrafish model expressing hCYPs can be readily prepared, unlike the preparation of transgenic fish. Although our zebrafish expressed hCYPs throughout the body, the developmental stage and organ in which hCYP is expressed can be selected by using other promoters such as an organ-specific promoter and heat-shock promoter. Moreover, zebrafish that express CYPs of a species other than humans can be prepared. Since the CYP subtype that is involved in bioactivation of a specific chemical is not always known in detail except for mutagenicity by an Ames test, our model can contribute to determination of the CYP subtype required for bioactivation and the mechanism of toxicological actions. Since rabbits are necessary in developmental toxicology testing, the establishment of transgenic zebrafish expressing hCYP3A7 is useful for screening tests as well as a research model.

## Data Availability

The datasets generated during and/or analyzed during the current study are available from the corresponding author on reasonable request.

## Supplementary Materials

The following supporting information can be downloaded at: https://www.mdpi.com/article/10.3390/ph16030368/s1, Figure S1: Effects of thalidomide on hCYP3A4-expressing zebrafish during development; Figure S2: Profiles of hCYP1A1-expressing zebrafish; Figure S3: No effect of thalidomide on fgf8 expression in the hyoid of hCYP3A7-expressing zebrafish; Figure S4: Effect of thalidomide on sall4 and shh expression in pectoral fin buds in hCYP3A7-expressing zebrafish; Figure S5: Map of the pT2A plasmid used for transgenesis of human CYPs (hCYPs) into the zebrafish genome; Figure S6: Criteria for severity of pectoral fin defects; Figure S7: Criteria for severity of pericardial edema; Table S1: Edema status in larvae with pectoral fin defects; Table S2: Nucleotide sequences of primers.
