# Supplementary material for: Augmentation of Pectoral Fin Teratogenicity by Thalidomide in Human Cytochrome P450 3A-Expressing Zebrafish
Source: Pharmaceuticals (Basel). 2023 Feb 28;16(3):368. doi: 10.3390/ph16030368 (PMC10055635; doi:10.3390/ph16030368)
Supplement: Supplementary file 1 [file pharmaceuticals-16-00368-s001.zip › pharmaceuticals-2218471-supplementary.pdf]

# Table S1 Edema status in larvae with pectoral fin defects

| Pectoral fin defects | | Edema | | |
|---|---|---|---|---|
| | | Severe | Mild | No |
| Unilateral | Shortening | 1 | | 3 |
| | Loss | 2 | | 1 |
| Bilateral | Shortening | 1 | 5 | 6 |
| | Loss | 2 | | |

Table S1. Edema status in larvae with pectoral fin defects. Embryos/larvae were exposed to 200 μM thalidomide from about 17 hpf, and pectoral fin defects and edema were observed at 72 hpf. Pectoral fin defects and edema status were determined according to the criteria shown in the legends of Figures S6 and S7. Two larvae with bilateral loss of pectoral fins showed severe edema and severe growth retardation.

# Figure S1 Effects of thalidomide on hCYP3A4-expressing zebrafish during development

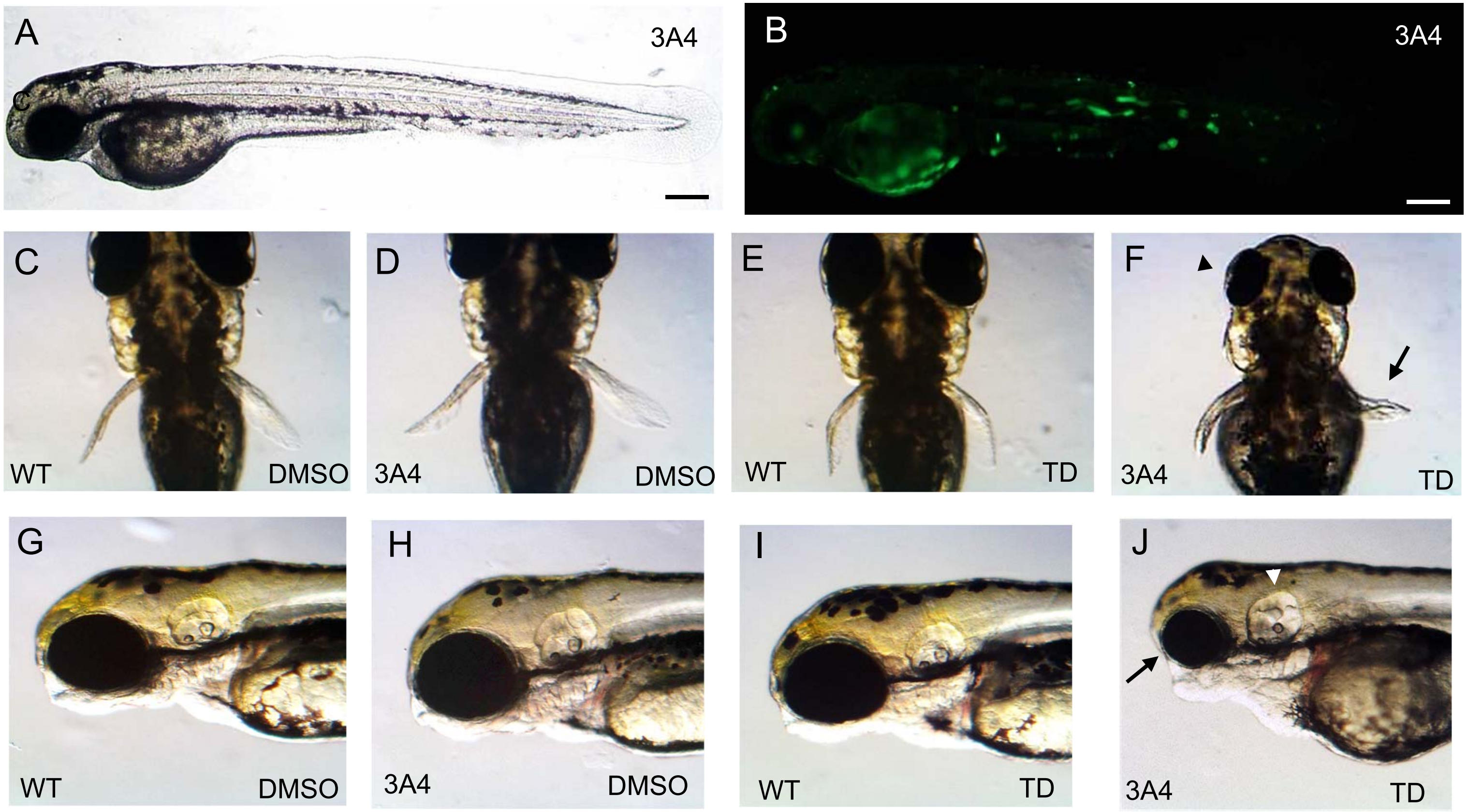


Figure S1. Effects of thalidomide on hCYP3A4-expressing zebrafish during development. Embryos/larvae of wild-type (WT) zebrafish (C, E, G and I) and hCYP3A4-expressing zebrafish (3A4) (A, B, D, F, H and J) were exposed to 200 µM thalidomide (TD) or 0.02% DMSO as a vehicle from 24 hpf to 72 hpf. B is an image of an EGFP/hCYP3A4-expressing embryo with its bright-field image (A) at 50 hpf. The arrow and arrowhead indicate a short pectoral fin and a small eye, respectively (F). The arrow and arrowhead indicate a small eye and an otic vesicle, respectively (J). Scale bars = 200 µm.

# Figure S2 Profiles of hCYP1A1-expressing zebrafish

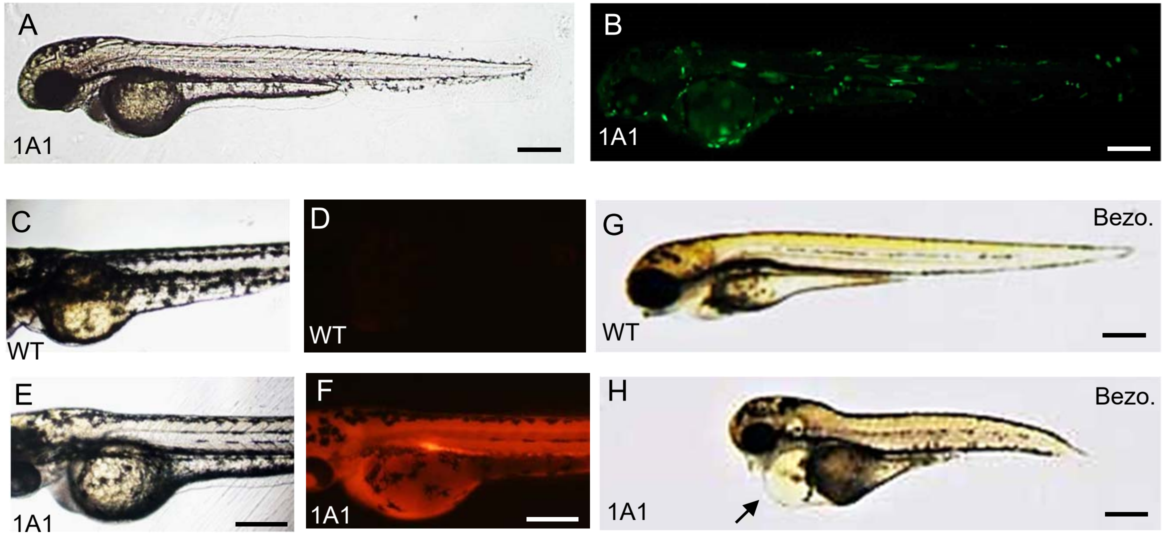


Figure S2. Profiles of hCYP1A1-expressing zebrafish. Fluorescent image of hCYP1A1-expressing zebrafish (hCYP1A1-zebrafish, 1A1) (B) and its bright field image at 50 hpf (A). Wild-type (WT) (C, D) and hCYP1A1-zebrafish (1A1) (E, F) were exposed to 7-ethoxyresorufin. D and F are fluorescent images of WT and hCYP1A1-zebrafish and C and E are their bright-field images. WT (G) and hCYP1A1-zebrafish (H) were exposed to 25 μM benzo[*a*]pyrene (Benzo.) from 24 hpf and were checked for their anomalies at 72 hpf. The arrow in H indicates pericardial edema. Scale bars = 200 μm.

# Figure S3 No effect of thalidomide on fgf8 expression in the hyoid of hCYP3A7-expressing zebrafish

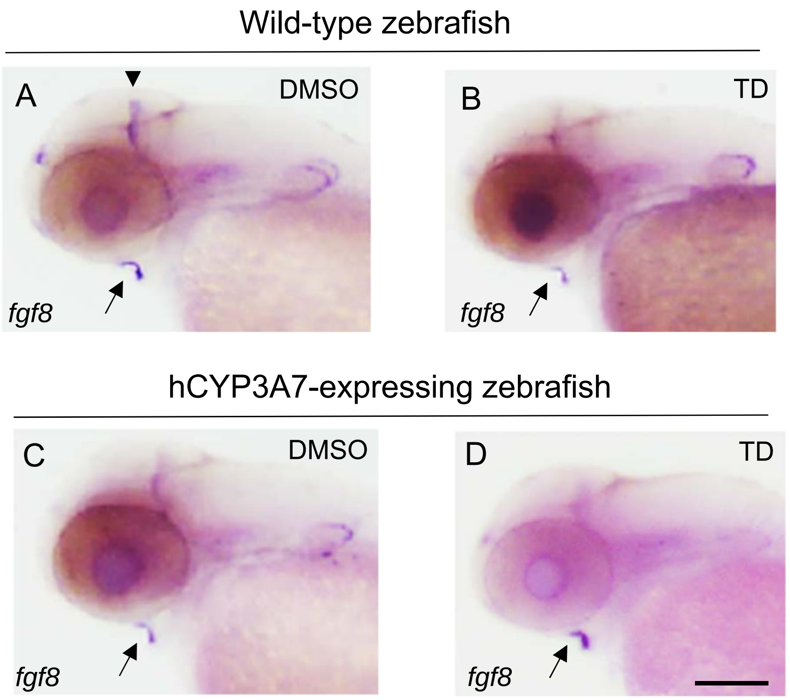


Figure S3. No effect of thalidomide on fgf8 expression in the hyoid of hCYP3A7-expressing zebrafish. Wild-type (A-B) and hCYP3A7-expressing embryos/larvae (C, D) were exposed to 200 µM thalidomide (TD) (B, D) or 0.02% DMSO (DMSO) (A, C) as a vehicle from 17 hpf until 48 hpf. Larvae were fixed at 48 hpf for whole-mount *in situ* hybridization with a probe of fgf8. Scale bar =100 µm. Representative images are shown (n= 24-30. Positive signals in the hyoid were observed in all embryos/larvae.

# Figure S4 Effect of thalidomide on sall4 and shh expression in pectoral fin buds in hCYP3A7-expressing zebrafish

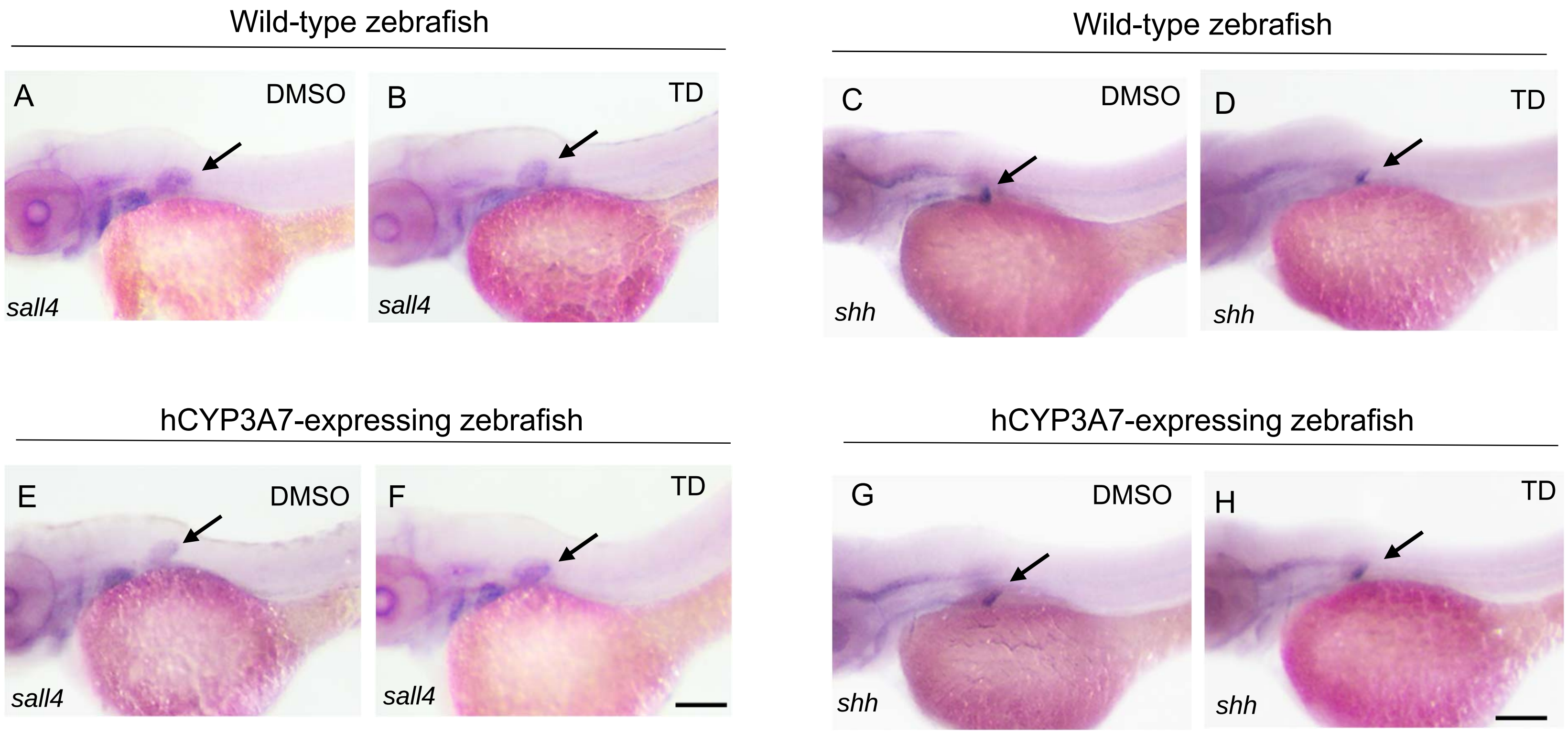


Figure S4. Effect of thalidomide on sall4 and shh expression in pectoral fin buds in hCYP3A7-expressing zebrafish. Wild-type (WT) (A-D) and hCYP3A7-expressing embryos/larvae (hCYP3A7-zebrafish)(E-H) were exposed to 200 µM thalidomide (TD) (B, D, F, H) or 0.02% DMSO (DMSO) (A, C, E, G) as a vehicle from 17 hpf until 48 hpf. Larvae were fixed at 48 hpf for whole-mount *in situ* hybridization with probes of sall4 and shh. Scale bars =100 µm. Representative images are shown (n=16). Positive signals in the pectoral fin buds found in 16 of 16 for sall4 (A, B, E, F) and for shh (C, D, G) except H (14/16).

# Figure S5 Map of the pT2A plasmid used for transgenesis of human CYPs (hCYPs) into the zebrafish genome

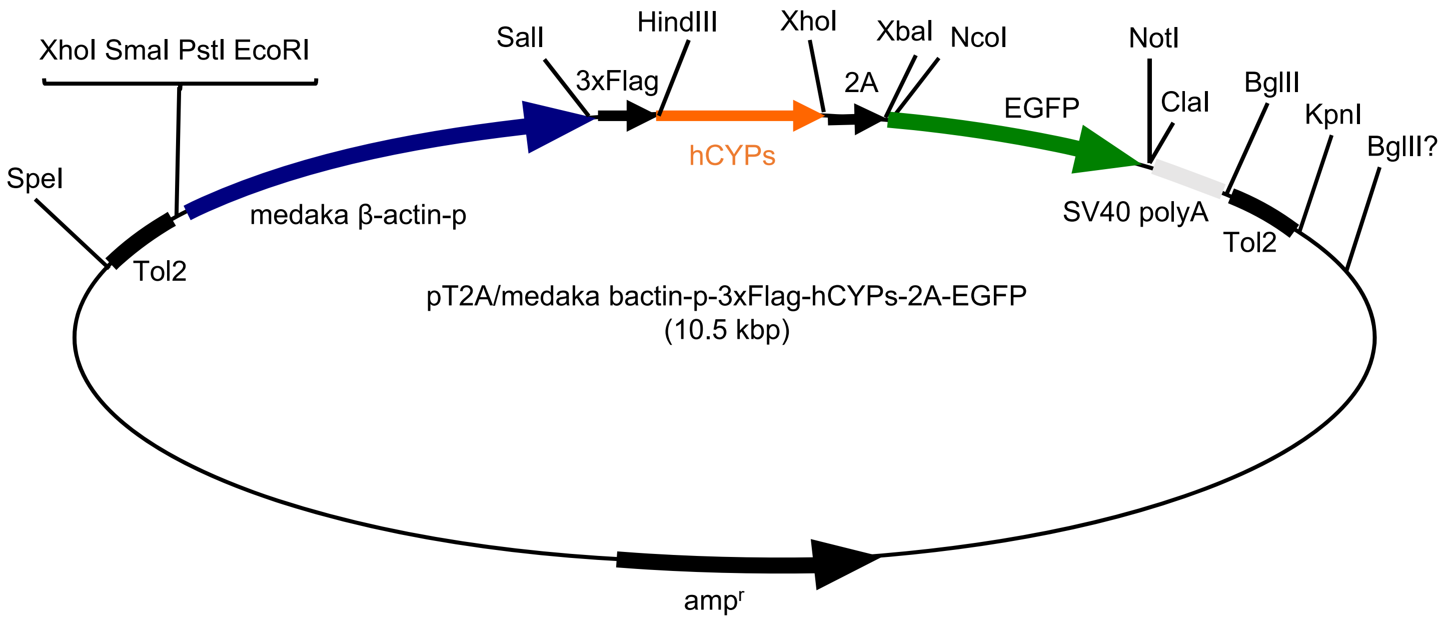


Figure S5. Map of the pT2A plasmid used for transgenesis of human CYPs (hCYPs) into the zebrafish genome.
A Tol2 transposon vector (pT2A) containing an open reading frame of hCYP-2A peptide-EGFP under the control of a medaka β-actin promoter (medaka β-action-p) was prepared.

# Table S2 Nucleotide sequences of primers

| Primer | Sequence | Purpose | Accession No. |
|---|---|---|---|
| hCYP3A7-F1 | 5’-GGAAAGTAGTGATGG-3’ | pT2A vector for transgenesis | NM_000765.5 |
| hCYP3A7-R1 | 5’-GGCTCCACTTACGGT-3’ | pT2A vector for transgenesis | NM_000765.5 |
| hCYP3A7-BamHI-F2 | 5’-CTCGGATCCGGAAAGTAGTGAT-3’ | pT2A vector for transgenesis | NM_000765.5 |
| hCYP3A7-Xho I-R2 | 5’-ATACTCGAGGGCTCCACTTACG-3’ | pT2A vector for transgenesis | NM_000765.5 |
| hCYP3A4-F1 | 5’-ATGGCTCTCATCCCAG-3’ | pT2A vector for transgenesis | DQ924960.1 |
| hCYP3A4-R1 | 5’-GGCTCCACTTACGGTG-3’ | pT2A vector for transgenesis | DQ924960.1 |
| hCYP3A4-SalI-F2 | 5’-ATTGTCGACATGGCTCTCATCCCAG-3’ | pT2A vector for transgenesis | DQ924960.1 |
| hCYP3A4-Xho I-R2 | 5’-ATTCTCGAGGGCTCCACTTACGGTG-3’ | pT2A vector for transgenesis | DQ924960.1 |
| hCYP1A1-F1 | 5’-ATGCTTTTCCCAATCTCC-3’ | pT2A vector for transgenesis | NM_001319216.2 |
| hCYP1A1-R1 | 5’-CTAAGAGCGCAGCTG-3’ | pT2A vector for transgenesis | NM_001319216.2 |
| hCYP1A1-Hind III-F2 | 5’-AAGCTTATGCTTTTCCCAATCTCC-3’ | pT2A vector for transgenesis | NM_001319216.2 |
| hCYP1A1-Xho I-R2 | 5’-CTCGAGCTAAGAGCGCAGCTG-3’ | pT2A vector for transgenesis | NM_001319216.2 |
| zfgf24-F | 5’-GTTCTGCCGTCAAGGTTCAT-3’ | Probe for WISH | NM_182871.3 |
| zfgf24-R | 5’-GCCCTTCGTGTCCTTTTCGT-3’ | Probe for WISH | NM_182871.3 |
| zsall4-F | 5’-ACTATCCCCACATCCCTTCC-3’ | Probe for WISH | NM_001080609.1 |
| zsall4-R | 5’-GAAGCTTTGCGGTACCAGAG-3’ | Probe for WISH | NM_001080609.1 |
| hCYP3A7-F | 5’-TCCAACATTCACCAGCGGAA-3’ | Probe for WISH | NM_000765.5 |
| hCYP3A7-R | 5’-TCATCACCACCACCCCTTTG-3’ | Probe for WISH | NM_000765.5 |

# Figure S6 Criteria for severity of pectoral fin defects

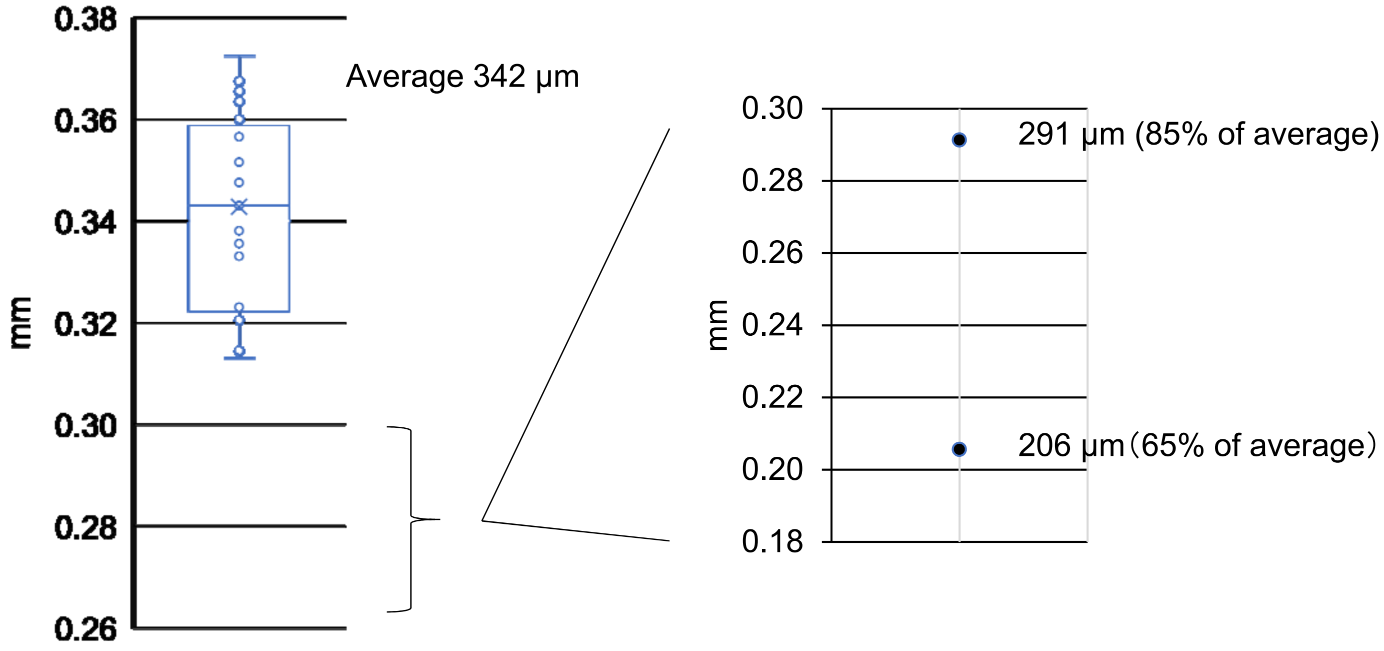


Figure S6. Criteria for severity of pectoral fin defects. The distribution of pectoral fin lengths of wild-type zebrafish at 72 hpf is indicated (n=29). If the length of a pectoral fin was less than 65% of the average value (<206 µm), it was judged as shortening, following Asatsuma-Okumura et al. [51].

# Figure S7 Criteria for severity of pericardial edema

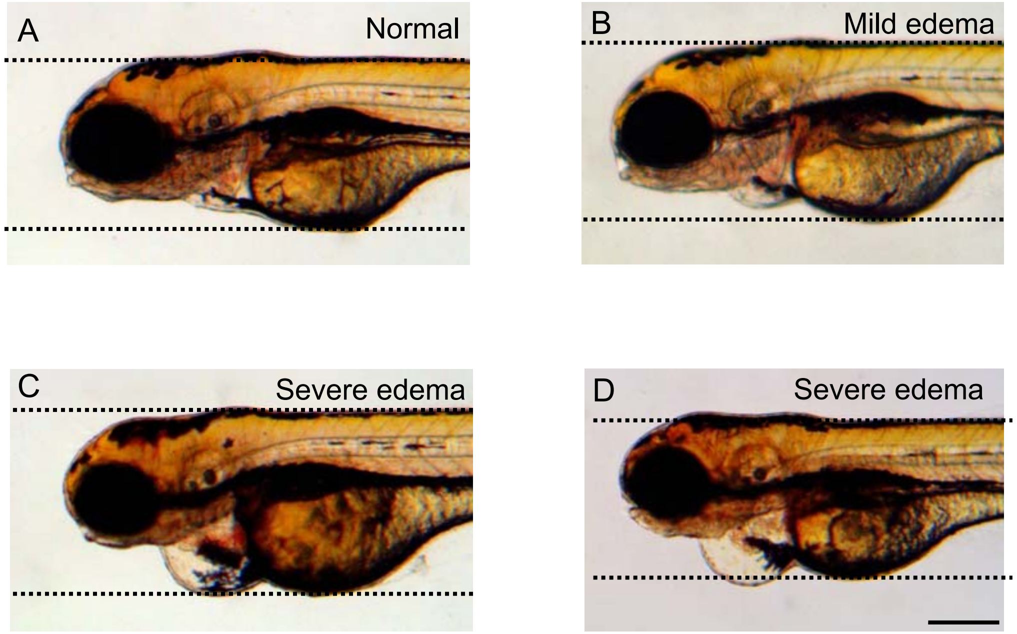


Figure S7. Criteria for severity of pericardial edema. Two horizontal lines (interrupted lines in A-D) beneath the back and yolk were drawn in lateral images of 72-hpf larvae. If the pericardial area was within these two interrupted lines, it was judged as mild edema (B). If the pericardial area was on or below the lower line, it was judged as severe edema (C, D). Scale bar = 200 μm.
